# Differences in ballistic findings between autopsy and post-mortem computed tomography in the head and neck region of gunshot victims: a comprehensive synthesis for forensic decision-making

**DOI:** 10.17179/excli2023-6256

**Published:** 2023-07-07

**Authors:** Carlos Antonio Vicentin-Junior, Raíssa Bastos Vieira, Nicole Prata Damascena, Melina Calmon Silva, Bianca Marques Santiago, Eugénia Cunha, João Paulo Mardegan Issa, Paulo Ricardo Martins-Filho, Carlos Eduardo Palhares Machado

**Affiliations:** 1 Graduate Program in Pathology, Ribeirão Preto Medical School, University of São Paulo, USP, Ribeirão Preto, Brazil; 2 Investigative Pathology Laboratory, Federal University of Sergipe, Sergipe, Brazil; 3 Graduate Program in Health Sciences, Federal University of Sergipe, Sergipe, Brazil; 4 National Center for the Dissemination of Forensic Sciences, Brazilian Federal Police, Distrito Federal, Brazil; 5 Graduate Program in Dentistry, Federal University of Paraiba, Paraiba, Brazil; 6 Centre for Functional Ecology, Laboratory of Forensic Anthropology, University of Coimbra, Portugal; 7 National Institute of Legal Medicine and Forensic Sciences, Lisbon, Portugal; 8 National Institute of Criminalistics, Brazilian Federal Police, Distrito Federal, Brazil

## ⁯

Firearms are frequently the primary cause of death in cases involving homicide, suicide, and unintentional injuries on a global scale (Naghavi et al., 2018[7]). In this context, forensic sciences play a crucial role in the criminal investigation process, providing scientific expertise to determine the circumstances, causes, and consequences of firearm-related incidents. The head and neck region is particularly susceptible to fatal firearm injuries, and the complex anatomical structures and intricate nature of injuries in this area necessitate meticulous examination and analysis to uncover the sequence of events leading to death.

While autopsy is widely regarded as the gold standard for post-mortem examination, there is growing recognition of the value of imaging techniques, such as computed tomography, in forensic ballistic investigations. Here, we investigated differences in ballistics findings between conventional autopsy and post-mortem computed tomography (PMCT) specifically in gunshot victims within the head and neck region. By synthesizing the best available evidence, this research aims to contribute to the ongoing discourse surrounding the application of PMCT and its potential implications for informed forensic decisions.

This systematic review and meta-analysis adhered to the Preferred Reporting Items for Systematic Reviews and Meta-Analyses (PRISMA) guideline. A comprehensive search of scientific evidence was conducted using various databases, including PubMed, Web of Science, Scopus, Embase, Google Scholar, and Open Access Theses and Dissertations. The search was carried out on June 7, 2023, employing a structured search strategy outlined in the supplementary file (Supplementary Table 1). Eligible studies had to report at least one of the following ballistic findings in the head and neck region: entrance/exit wounds, wound track, or projectile fragments. We excluded case reports, small case series with fewer than 10 cases, and studies with potential overlapping populations.

The assessment of search results was conducted independently by two reviewers using a two-stage process. Initially, titles and abstracts were reviewed, and potentially relevant studies were identified for a full-text evaluation. Any disagreements between the reviewers were resolved through a consensus-based approach or with the assistance of a third reviewer. Using a standardized data extraction form, we recorded data on authorship, publication year, study country, sample size and characteristics, details of the examinations conducted on gunshot victims, and ballistic findings. The Joanna Briggs Institute Critical Appraisal Checklist for Analytical Cross-Sectional Studies (https://jbi.global/critical-appraisal-tools) was used to assess the risk of bias. 

To ensure a robust and accurate analysis, we followed standard statistical procedures in our study. First, we calculated the prevalence of each ballistic finding in the head and neck region, comparing results from both PMCT and autopsy. Subsequently, we utilized a random-effects model to synthesize prevalence ratios (PR) with 95 % confidence intervals (CI) and visually presented them using forest plots. We assessed the degree of heterogeneity between studies using the I^2^ index. The significance level of 5 % was used to determine the statistical significance. All analyses were conducted using the R software (version 3.5.3; R Foundation for Statistical Computing, Vienna, Austria).

The initial search yielded 704 references. After screening the titles and abstracts, 31 full-text articles were assessed for eligibility and seven studies (Levy et al., 2006[6]; Andenmatten et al., 2008[1]; Kirchhoff et al., 2016[5]; Elkhateeb et al., 2018[2]; Graziani et al., 2018[3]; van Kan et al., 2019[10]; Ursprung et al., 2022[9]) met the inclusion criteria (supplementary file; Supplementary Figure 1). These studies involved a total of 203 gunshot victims. Data on ballistic outcomes, including projectile entrance/exit injuries (Andenmatten et al., 2008[1]; Elkhateeb et al., 2018[2]; van Kan et al., 2019[10]; Ursprung et al., 2022[9]), wound track (Levy et al., 2006[6]; Kirchhoff et al., 2016[5]; Elkhateeb et al., 2018[2]; Graziani et al., 2018[3]; van Kan et al., 2019[10]; Ursprung et al., 2022[9]), and detection of projectile fragments (Elkhateeb et al., 2018[2]; Graziani et al., 2018[3]; van Kan et al., 2019[10]; Ursprung et al., 2022[9]) were compiled for analysis in this study (supplementary file; Supplementary Table 2). Additional details on the method of image acquisition, processing, and visualization can be found in the supplementary file (supplementary Table 3). Overall, the included studies were assessed as having a low risk of bias (supplementary file; Supplementary Figure 2).

We observed no significant differences in ballistic findings for entrance/exit wounds (PR 0.81, 95 % CI 0.61 - 1.09; p = 0.162; I^2^ = 44.1 %) and wound track (PR 1.00, 95 % CI 0.86 - 1.16; p = 0.180; I^2^ = 0 %) between PMCT and autopsy However, PMCT had a 26 % higher prevalence of projectile fragments found than conventional autopsy (PR 1.26, 95 % CI 0.99 - 1.60; p = 0.056; I^2^ = 0 %) (supplementary file; Supplementary Figure 3).

The findings of this meta-analysis revealed that PMCT was more effective than autopsy in detecting a higher frequency of projectile fragments in the head and neck region. This finding is of utmost significance due to the challenges faced during autopsy examinations, where the cranial vault is typically the only area opened, leaving the face intact (Oehmichen et al., 2003[8]; Jalalzadeh et al., 2015[4]). The limited access to the head and neck region during autopsy can result in a lower detection rate of projectile fragments. The use of PMCT overcomes these limitations by providing detailed and non-invasive 3D imaging, allowing for enhanced visualization and detection of projectiles in this challenging anatomical region. The detection of projectile fragments in the head and neck region can provide critical information on the direction of the shot, type of firearm, and the distance from which the shot was fired, thereby aiding in criminal investigations, and providing valuable evidence for legal proceedings. Therefore, the implementation of CT in routine forensic practice is suggested for cases involving firearm-related deaths, especially for the detection of projectile fragments in the head and neck region.

While PMCT has demonstrated its advantages in detecting projectile fragments in the head and neck region, it is important to note that this technique should be considered as a complementary tool rather than a replacement for conventional autopsy. The combination of both approaches can provide a comprehensive and accurate assessment of ballistic findings in gunshot victims. Furthermore, the incorporation of other emerging imaging techniques and the expertise of experienced forensic radiologists, may further enhance the detection and analysis of ballistic findings.

In conclusion, this study provides important evidence regarding the similarities and differences in ballistic findings between PMCT and conventional autopsy in gunshot victims. The superior detection of projectile fragments in the head and neck region through PMCT highlights its potential as a valuable adjunct to conventional autopsy. However, further research and standardization are needed to fully utilize the benefits of PMCT and optimize its integration into routine forensic practice. By addressing these research gaps and challenges, the forensic community can improve the accuracy and efficiency of investigations into firearm-related deaths, thereby contributing to the pursuit of justice.

## Declaration

### Conflict of interest

The authors declare no conflict of interest.

### Acknowledgments

This study was conducted under the auspices of the ANP/CNPq Forensic Anthropology and Identification of Persons Research Group. CAVJ and RBV received a master's scholarship from CAPES (Coordenação de Aperfeiçoamento de Pessoal de Nível Superior), Brazil. 

## Supplementary Material

Supplementary information
