# Supplementary material for: Differences in ballistic findings between autopsy and post-mortem computed tomography in the head and neck region of gunshot victims: a comprehensive synthesis for forensic decision-making
Source: EXCLI J. 2023 Jul 7;22:600–3. doi: 10.17179/excli2023-6256 (PMC10471839; doi:10.17179/excli2023-6256)
Supplement: Supplementary information [file EXCLI-22-600-s-001.pdf]

*EXCLI Journal 2023;22:600-603 – ISSN 1611-2156*
*Received: June 16, 2023, accepted: July 03, 2023, published: July 07, 2023*

## Supplementary information to:

## Letter to the editor:

# DIFFERENCES IN BALLISTIC FINDINGS BETWEEN AUTOPSY AND POST-MORTEM COMPUTED TOMOGRAPHY IN THE HEAD AND NECK REGION OF GUNSHOT VICTIMS: A COMPREHENSIVE SYNTHESIS FOR FORENSIC DECISION-MAKING

Carlos Antonio Vicentin-Junior[1,2], Raíssa Bastos Vieira[1,2], Nicole Prata Damascena[2,3], Melina Calmon Silva[4], Bianca Marques Santiago[5], Eugénia Cunha[6,7], João Paulo Mardegan Issa[1], Paulo Ricardo Martins-Filho[2,3,*], Carlos Eduardo Palhares Machado[1,4,8]

[1] Graduate Program in Pathology, Ribeirão Preto Medical School, University of São Paulo, USP, Ribeirão Preto, Brazil
[2] Investigative Pathology Laboratory, Federal University of Sergipe, Sergipe, Brazil
[3] Graduate Program in Health Sciences, Federal University of Sergipe, Sergipe, Brazil
[4] National Center for the Dissemination of Forensic Sciences, Brazilian Federal Police, Distrito Federal, Brazil
[5] Graduate Program in Dentistry, Federal University of Paraiba, Paraiba, Brazil
[6] Centre for Functional Ecology, Laboratory of Forensic Anthropology, University of Coimbra, Portugal
[7] National Institute of Legal Medicine and Forensic Sciences, Lisbon, Portugal
[8] National Institute of Criminalistics, Brazilian Federal Police, Distrito Federal, Brazil

* **Corresponding author:** Paulo Ricardo Martins-Filho, Universidade Federal de Sergipe, Hospital Universitário, Laboratório de Patologia Investigativa. Rua Cláudio Batista, s/n. Sanatório. Aracaju, Sergipe, Brazil. CEP: 49060-100. E-mail: prmartinsfh@gmail.com

https://dx.doi.org/10.17179/excli2023-6256

This is an Open Access article distributed under the terms of the Creative Commons Attribution License (http://creativecommons.org/licenses/by/4.0/).

*EXCLI Journal 2023;22:600-603 – ISSN 1611-2156*
*Received: June 16, 2023, accepted: July 03, 2023, published: July 07, 2023*

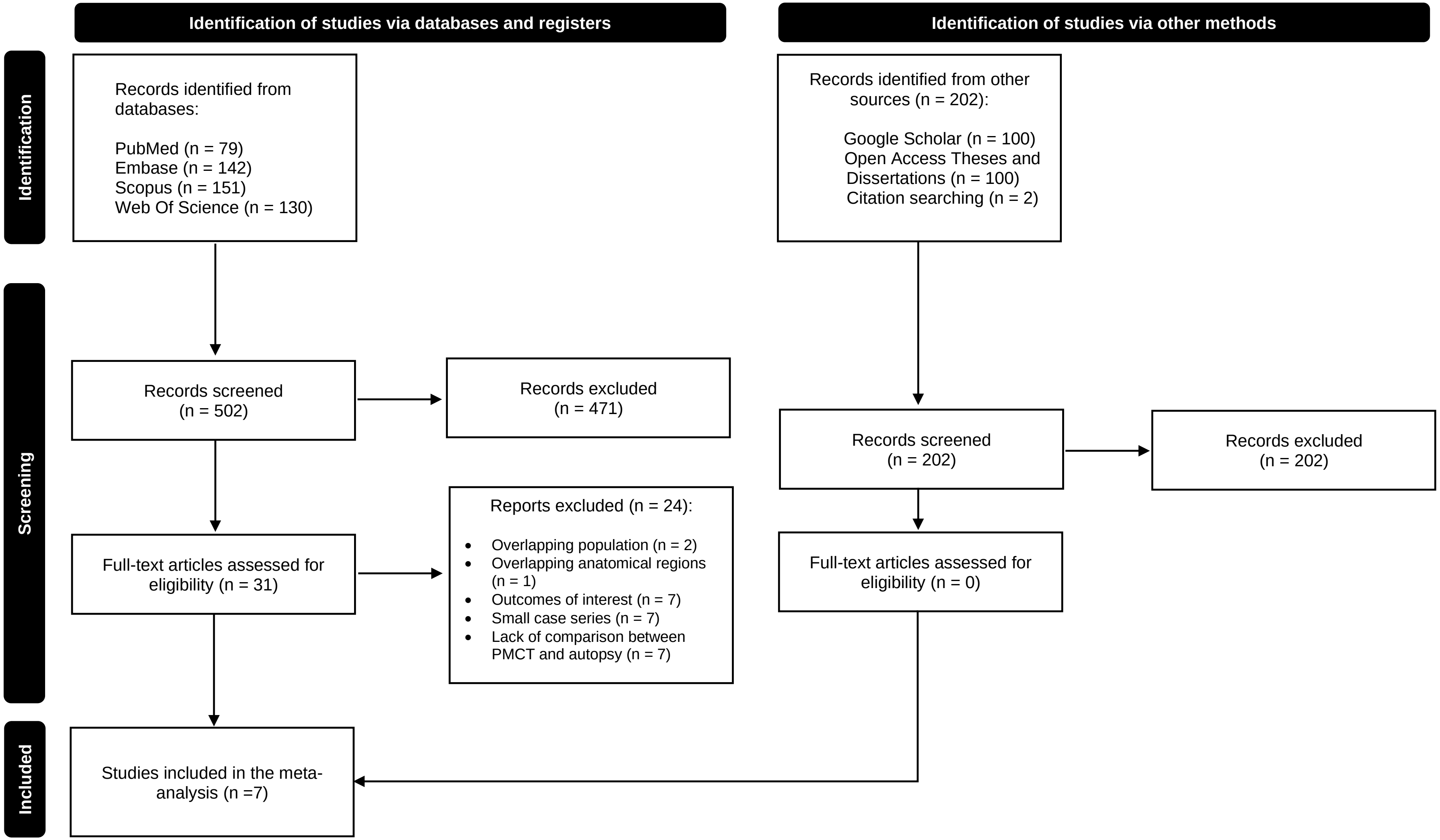


**Supplementary Figure 1:** Flow diagram of study selection

*EXCLI Journal 2023;22:600-603 – ISSN 1611-2156*
*Received: June 16, 2023, accepted: July 03, 2023, published: July 07, 2023*

| Question | Author | | | | | | |
|---|---|---|---|---|---|---|---|
| | Andenmatten *et al.* 2008 | Elkhateeb *et al.* 2018 | Graziani *et al.* 2018 | Kirchhoff *et al.* 2016 | Levy *et al.* 2006 | Ursprung *et al.* 2022 | van Kan, *et al.* 2019 |
| **1.** Were the criteria for inclusion in the sample clearly defined? | ✗ | ✓ | ✓ | ✗ | ✓ | ✓ | ✓ |
| **2.** Were the study subjects and the setting described in detail? | ✓ | ✓ | ✓ | ✓ | ✓ | ✓ | ✗ |
| **3.** Was the exposure measured in a valid and reliable way? | ✓ | ✓ | ✓ | ✓ | ✓ | ✓ | ✓ |
| **4.** Were objective, standard criteria used for measurement of the condition? | ✓ | ✓ | ✓ | ✓ | ✓ | ✗ | ✓ |
| **5.** Were confounding factors identified? | ? | ? | ? | ? | ? | ? | ? |
| **6.** Were strategies to deal with confounding factors stated? | ? | ? | ? | ? | ? | ? | ? |
| **7.** Were the outcomes measured in a valid and reliable way? | ✓ | ✓ | ✓ | ✓ | ✓ | ✓ | ✓ |
| **8.** Was appropriate statistical analysis used? | ✓ | ✓ | ✓ | ✓ | ✓ | ✓ | ✓ |

**Supplementary Figure 2:** Risk of bias assessment

*EXCLI Journal 2023;22:600-603 – ISSN 1611-2156*
*Received: June 16, 2023, accepted: July 03, 2023, published: July 07, 2023*

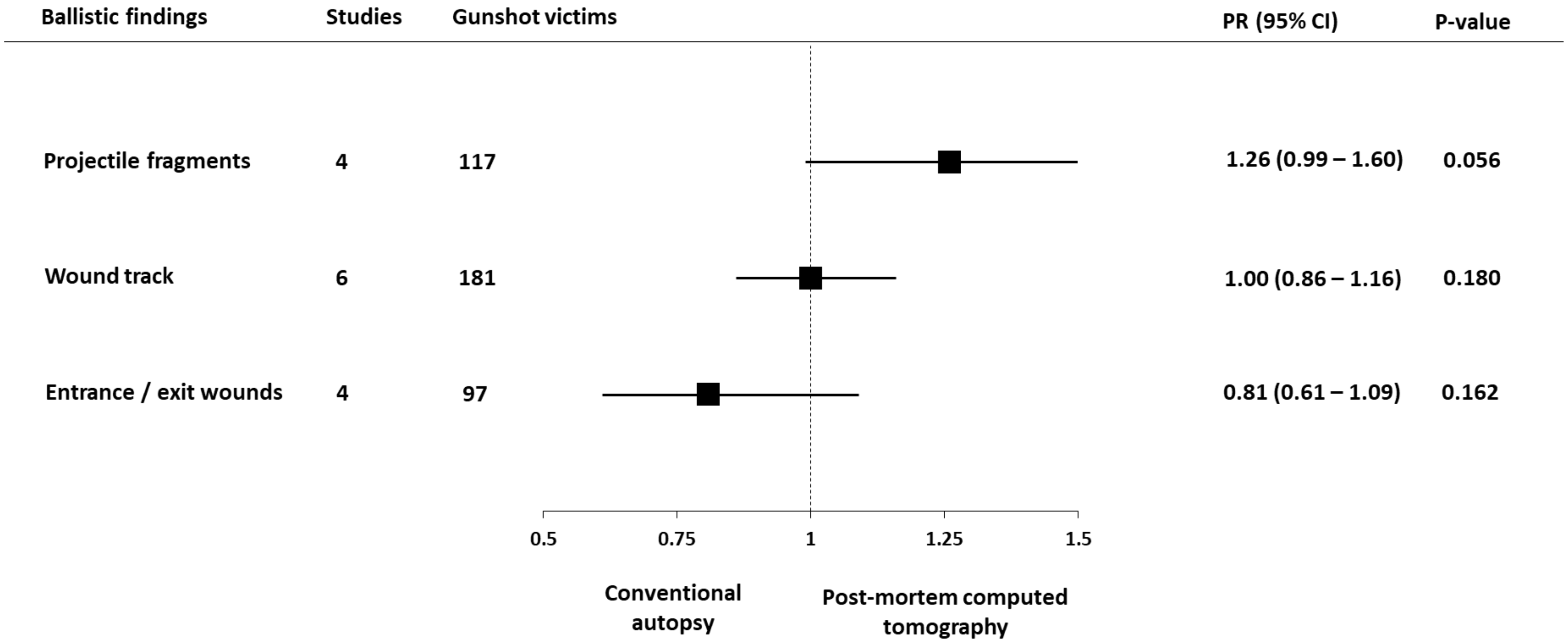


**Supplementary Figure 3:** Differences between post-mortem computed tomography and conventional autopsy in the head and neck region regarding projectile fragments, wound track, and entrance / exit wounds

*EXCLI Journal 2023;22:600-603 – ISSN 1611-2156*
*Received: June 16, 2023, accepted: July 03, 2023, published: July 07, 2023*

**Supplementary Table 1:** Search strategy

| Databases and other sources | Search strategy |
|---|---|
| **PubMed**<br>**Web of Science**<br>**Scopus**<br>**Embase**<br>**Google Scholar**<br>**Open Access Theses and Dissertations** | (“virtual autopsy” OR virtopsy OR “touch-free autopsy” OR “digital autopsy” OR “scalpel-free autopsy” OR “postmortem CT” OR “postmortem computed tomography” OR “postmortem CT” OR “postmortem computed tomography” OR “post-mortem CT” OR “post-mortem computed tomography” OR PMCT OR “robotic machine” OR virtibot OR “computed tomography virtopsy”) AND (gun OR gunshot OR gunshots OR gunfire OR shooting OR firearm OR firearms OR weapon OR weapons OR handgun OR handguns) |

**Supplementary Table 2:** Characteristics of the included studies

| Author | Year | Country | Sample size | Sex | | Ballistic outcomes | | |
|---|---|---|---|---|---|---|---|---|
| | | | | Male | Female | Entrance/exit wounds | Wound track | Projectile fragments |
| Andenmatten et al. | 2008 | Switzerland | 22 | 14 | 8 | Yes | NR | NR |
| Elkhateeb et al. | 2018 | Egypt | 30 | 24 | 6 | Yes | Yes | Yes |
| Graziani et al. | 2018 | Israel | 42 | NR | NR | NR | Yes | Yes |
| Kirchhoff et al. | 2016 | Germany | 51 | 40 | 11 | NR | Yes | NR |
| Levy et al. | 2006 | USA | 13 | 13 | 0 | NR | Yes | NR |
| Ursprung et al. | 2022 | Switzerland | 24 | 19 | 5 | Yes | Yes | Yes |
| van Kan et al. | 2019 | Netherlands | 21 | NR | NR | Yes | Yes | Yes |

NR, Not reported

EXCLI Journal 2023;22:600-603 – ISSN 1611-2156
Received: June 16, 2023, accepted: July 03, 2023, published: July 07, 2023

**Supplementary Table 3:** Method of image acquisition, processing, and visualization

| Author | Equipment | kV | mA | mAs | Acquisition time | Image reconstruction or visualization software |
|---|---|---|---|---|---|---|
| Andenmatten et al., 2008 | Somatom Emotion 6 (Siemens Medical Solutions) | NR | NR | NR | < 10 minutes | Syngo CT work-station (Siemens Medical Solutions) |
| Elkhateeb et al., 2018 | Somatom Sensation Cardiac 64 (Siemens Medical Solutions) | NR | NR | NR | NR | Syngo B work-station (Siemens Medical Solutions) |
| Graziani et al., 2018 | Toshiba Aquilion PRIME scanner or Phillips iCT 256 Scanner | 120 (under 100kg)<br>135 (over 100 kg) | 350 (Torso)<br>350 (Head)<br>400 (Neck) | 430 (Torso)<br>480 (Neck)<br>840 (Head) | NR | Phillips Portal or Vital-images Vitrea |
| Kirchhoff et al., 2016 | Brilliance 64 (Phillips) or GE Discovery 750 HD (GE Healthcare) | NR | NR | NR | NR | Picture Archiving Computer System |
| Levy et al., 2006 | LightSpeed 16 (GE Medical Systems) | NR | NR | NR | NR | Advantage (GE Medical Systems) |
| Ursprung et al., 2022 | Somatom Definition Flash (Siemens Medical Solutions) | 120 | NR | NR | NR | Leonardo work-station (Siemens Medical Solutions) and Picture Archiv-ing Computer System |
| van Kan et al., 2019 | NR | 120 | NR | 350 (Head)<br>300 (Neck)<br>450 (Torso) | NR | OsiriX |

kV, kilovoltage; mA, milliampere; mAs; milliampere-seconds; NR, not reported
